# Intra-articular Hyaluronic Acid in Treating Knee Osteoarthritis: a PRISMA-Compliant Systematic Review of Overlapping Meta-analysis

**DOI:** 10.1038/srep32790

**Published:** 2016-09-12

**Authors:** Dan Xing, Bin Wang, Qiang Liu, Yan Ke, Yuankun Xu, Zhichang Li, Jianhao Lin

**Affiliations:** 1 Arthritis Clinic & Research Center, Peking University People’s Hospital, Peking University, Beijing, China

## Abstract

Numerous meta-analyses have been conducted aiming to compare hyaluronic acid (HA) and placebo in treating knee osteoarthritis (OA). Nevertheless, the conclusions of these meta-analyses are not in consistency. The purpose of the present study was to perform a systematic review of overlapping meta-analyses investigating the efficacy and safety of HA for Knee OA and to provide treatment recommendations through the best evidence. A systematic review was conducted based on the PRISMA guidelines. The meta-analyses and/or systematic reviews that compared HA and placebo for knee OA were identified. AMSTAR instrument was used to evaluate the methodological quality of individual study. The information of heterogeneity within each variable was fetched for the individual studies. Which meta-analyses can provide best evidence was determined according to Jadad algorithm. Twelve meta-analyses met the eligibility requirements. The Jadad decision making tool suggests that the highest quality review should be selected. As a result, a high-quality Cochrane review was included. The present systematic review of overlapping meta-analyses demonstrates that HA is an effective intervention in treating knee OA without increased risk of adverse events. Therefore, the present conclusions may help decision makers interpret and choose among discordant meta-analyses.

Osteoarthritis (OA) is a common degenerative disorder with rising prevalence. It leads to cause of disability among the older people^1^,^2^,^3^. In epidemiology, half of the world’s population aged 65 years or older has OA, which is the most prevalent disorder of articulating joints in humans. Knee OA is the most common type of OA. The symptoms of knee OA is characterized by pain and disability in joints. In pathologically, the following features are in knee joints: damage of articular cartilage at weight-bearing areas, change in subchondral bone, inflammation in synovitis, osteophyte formation, cyst formation and thickening of joint capsule and loss of joint space^4^. As some evidence showed, the significant risk factors for this excess mortality in OA included walking disability and cardiovascular disorder^5^. Thus, more attention should be paid to alleviation of pain and improvement of joint function in OA patients.

Hyaluronic acid (HA) is, as an integral component of synovial fluid, often used in clinical practice for treating knee OA. HA is regarded as a joint lubricant during shear stress and as a shock absorber during compressive stress. In the development of knee OA, a marked reduction in concentration and molecular weight of endogenous HA ultimately leads to reduced viscoelastic properties of synovial fluid and induction of proinflammatory pathways^6^. Therefore, the purpose of intra-articular injection of exogenous HA is to replace this OA-induced deficit and stimulate production of endogenous HA^7^. HA may alleviate symptoms of knee OA via multiple pathways including inhibition of chondrodegradative enzymes and inflammatory processes, stimulation of chondrocyte metabolism, and synthesis of articular cartilage matrix components^8^.

Although numerous meta-analyses have been conducted to determine the safety and efficacy of HA injections for knee OA, they showed different results in their studies^9^,^10^,^11^,^12^. In the recent guideline in treating Knee OA, AAOS reported that HA is not recommended in the treatment of Knee OA^13^. However, Altman *et al*.^14^ investigated ten guidelines regarding the use of HA for the treatment of knee OA and reported that the recommendations were highly inconsistent as a result of the variability in guideline methodology. Thus, the inconsistent recommendations make it difficult for clinical professionals to determine its appropriateness when treating knee OA.

The purpose of the present study is to perform a systematic review of overlapping meta-analyses determining the clinical effects of HA in treating Knee OA, to evaluate the mythological quality of included individual meta-analyses, and to take best evidence through the currently inconsistent evidence.

## Materials and Methods

### Search strategy

The present systematic review was conducted following the guideline of PRISMA (Preferred Reporting Items for Systematic Reviews and Meta-analysis) statement^15^. PRISMA statement was used to guarantee high-quality reporting of systematic reviews or meta-analyses^16^. Electronic databases including MEDLINE, EMBASE and Cochrane library were searched for all meta-analysis or systematic review published through Nov 2015. The following MeSH items or free words were taken: osteoarthritis, knee, meta-analysis, systematic review, and hyaluronic acid. The references of searched studies were also reviewed to explore other meta-analyses or systematic reviews. No restrictions were made on the publication language.

### Inclusive and exclusive criteria

Studies were considered eligible for inclusion if they met the following criteria:

(1) Meta-analyses or systematic reviews only including randomized controlled trial (RCT);

(2) Meta-analyses or systematic reviews comparing HA with placebo in treating knee OA;

(3) Meta-analyses or systematic reviews reported at least one variable (such as pain, function, and safety).

Exclusion criteria included the following items:

(1) Meta-analyses or systematic reviews including non-RCT;

(2) Systematic reviews did not conducting meta-analysis or pooling data;

(3) Abstract, commentary, methodological study, narrative review.

### Meta-analyses/systematic reviews selection

Firstly, two reviewers assessed the titles and abstracts of researched studies for the eligibility criteria independently. The two reviewers were not blinded to the journals, organizations, financial assistance, conflict of interest and researchers’ information. Subsequently, the full text of the studies that potentially met the inclusion criteria was read to determine the final inclusion. Any disagreement was resolved by reaching a consensus through discussion.

### Date extraction

Two reviewers independently extracted the data from each included literature by the use of a standard data extraction form. The following items were extracted: title, authors, original study design, database, total number of studies, level of evidence, the pooled results and methodological variables.

### Assessment of methodological quality

The quality assessment was independently conducted by two authors. Disagreements were resolved by discussion or a third reviewer was involved. The Assessment of Multiple Systematic Reviews (AMSTAR) method was used to evaluate the methodological quality of included studies^17^. The AMSTAR was a measurement scale containing eleven items, and it was applied extensively in assessing methodological quality of published meta-analysis or systematic review^18^.

### Heterogeneity within included studies

Heterogeneity of each outcome (primary and secondary outcomes) was reported for the each included meta-analyses. The following two questions were also evaluated: whether sensitivity analysis was performed in meta-analysis and whether the included meta-analyses evaluated potential sources of heterogeneity across primary studies. Upon the Cochrane Handbook, Heterogeneity of each outcome between 0% and 40% is regarded as not important; between 30% and 60% is moderate; between 50% and 90% is substantial, and between 75% and 100% is considerable. Therefore, I^2^ less than 60% are accepted in the present study.

### Choice of best evidence

Treatment recommendations were made according to the Jadad decision algorithm^19^. The methodological instrument confirmed the source of inconsistence between meta-analyses, including differences in clinical problem, inclusion and exclusion standard, extracted data, methodological quality assessment, data combining, and statistical analysis methods^19^. The application of algorithm was performed by two independent reviewers. Our evaluation group came to conformity as to which of included meta-analyses can provide best evidence based on the current information.

## Results

### Literature search

**Figure 1:**
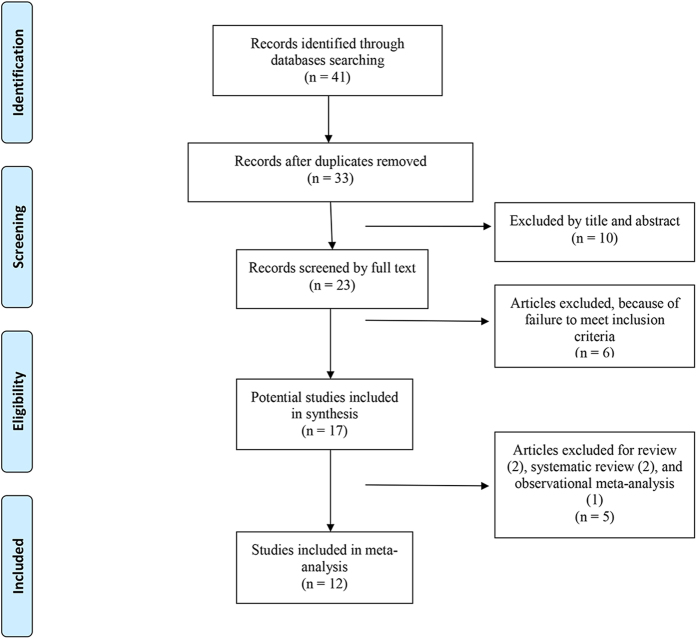
Flowchart of the study selection process.

Thirty-three titles and abstracts were preliminarily identified with the first search strategy, of which 12 of the published meta-analyses^6^,^9^,^10^,^11^,^12^,^20^,^21^,^22^,^23^,^24^,^25^,^26^ ultimately met the eligibility criteria (Fig. 1). Two studies^27^,^28^ were excluded because they conducted network meta-analysis among each kinds of HA. Three meta-analyses^29^,^30^,^31^ were excluded because they were performed to compare the efficacy and safety of HA with corticosteroids. Three studies, including primary studies in ankle/hip joints, were also excluded^32^,^33^,^34^.

Table 1 presented the characteristics of included meta-analysis. The number of original studies in meta-analysis varied from 5 in that study published in 2006 to 89 that published in 2012 (Table 2). All included meta-analyses conducted qualitatively data synthesis.

### Search methodology

The literature search methodology which was adopted by included meta-analysis was present in Table 3. Most of the databases that the included studies searched were Medline, Embase or Cochrane database.

### Methodological quality of included meta-analyses

Methodological characteristics of included Meta-analyses were presented in Table 4. All included meta-analyses only included RCTs and/or quasi-RCTs. The evidence degree of each meta-analysis was Level II. REVMAN, STATA, SAS, R and Comprehensive Meta-analysis software were used in meta-analyses. Subgroup and sensitivity analysis were used in some of the included studies. None meta-analysis used GRADE in their study. The AMSTAR results with each question of included meta-analysis were shown in Table 5. The average score of AMSTAR of included meta-analyses was 7.25, ranging from 4 to 11. All included meta-analyses reported that there was no conflict of interest in making meta-analysis. One meta-analysis conducted by Bellamy *et al*.^25^ was the highest quality study.

### Heterogeneity Assessment

Table 6 presented the data of heterogeneity of each variable in each meta-analysis. The I^2^ value was adopted to calculate the heterogeneity among original studies as a measurement aiming to ascertain the inter-studies variability in all included meta-analyses.

### Results of Jadad Decision Algorithm

**Figure 2:**
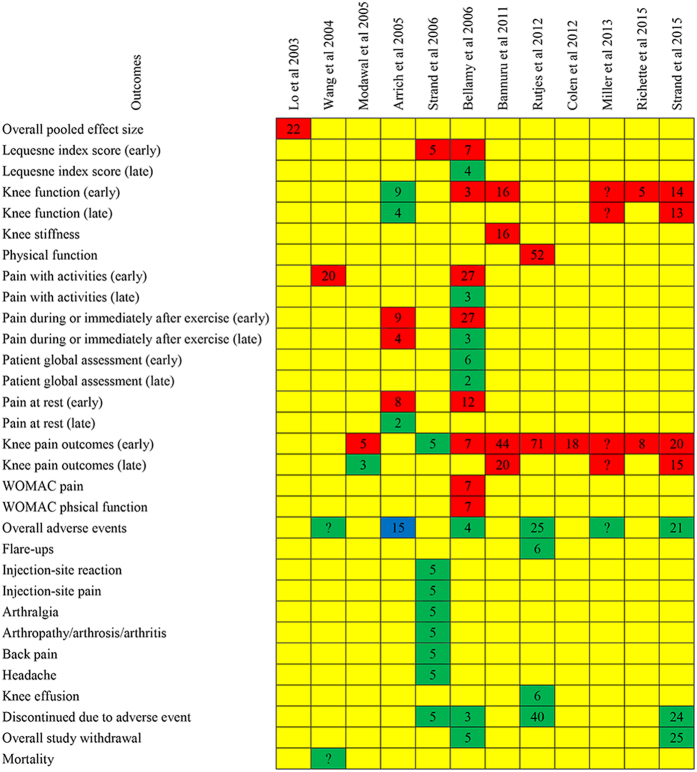
Results of each included meta-analysis. Red means favoring hyaluronic acid; green means no difference; yellow means not reporting; and blue means favoring placebo. Arabic numerals mean the number of included randomized clinical trials.

**Figure 3:**
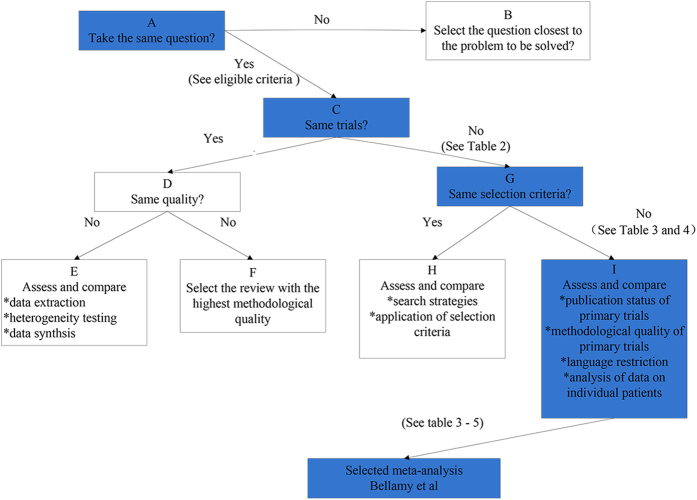
Flow diagram of Jadad decision algorithm.

All outcomes reported in primary meta-analyses were reported in Fig. 2. According to the following three respects (the meta-analyses addressed the same clinical question, did not include the same original studies, and not have similar inclusion/exclusion criteria), the Jadad algorithm proposed that the eligible meta-analyses can be elected on account of the methodological quality and publication statue (Fig. 3). As a result, a Cochrane meta-analysis^25^ with highest quality was selected. Bellamy *et al*. supported the use of the HA in the treatment of knee OA with beneficial effects on pain, function and patient global assessment.

## Discussion

According to the above mentioned methodology, the meta-analysis conducted by Bellamy *et al*.^25^ is with highest quality compared with others. The best available evidence hints that HA is an effective intervention in treating knee OA without increased risk of adverse events. Therefore, the current evidence supports the use of the HA in the treating knee OA.

Meta-analyses or systematic reviews are commonly regarded as the highest level of clinical evidence^35^. Clinicians can make meaningful clinical decisions with the help of meta-analyses or systematic reviews. However, a larger number of meta-analyses involving in the same clinical question have been published with conflicting results. This phenomenon was also occurred in the evidence-based study in HA injections for knee OA. Although numerous meta-analyses or systematic reviews have been written in treating knee OA via HA, there was still in controversy. Such discrepancy results in some difficulties for decision makers (including clinicians, policymakers and patients, depending on the context) who rely on this synthesized evidence to help them make decisions among pharmacological interventions when the results of trials are not unanimous.

Jadad *et al*.^19^ concluded the following potential sources of inconsistency among meta-analyses, including the clinical topic, eligible criteria, data extraction, quality assessment, assessment of the ability to combine studies, and statistical methods for data synthesis. Furthermore, Jadad *et al*.^19^ provided a decision methodological tool which summarizes the process for identifying and resolving causes of discordance. The ultimate purpose was to help clinical decision makers to select best evidence from inconsistency meta-analyses and systematic reviews. As recommended by Jadad *et al*., decision algorithm, a widely used tool^36^,^37^,^38^, is a useful instrument for differencing between meta-analyses or systematic reviews. Although Jadad decision algorithm choose comprehensive meta-analysis among discordant reviews, more empirical evidence is required to establish the effect of these elements on the validity of the review process, their relative importance and their effect on the results of a review.

According to the decision algorithm, the Cochrane meta-analysis conducted by Bellamy *et al*. was selected in the present study. Bellamy *et al*.^25^ reported that HA was an effective treatment for knee OA at different post injection periods but especially at the 5 to 13 week post injection period, and few adverse events were reported in the HA. However, there is considerable between-product, between-variable and time-dependent variability in the clinical response. Therefore, we concluded that HA is an effective and safety intervention in treating knee OA. Although the positive results were reported, effect size statistic was not used in the study. Thus, we did not have entire confidence in the extent of symptomatic improvement.

Rutjes and his colleagues^10^ published a high-quality systematic review (AMSTAR score: 10) in *Annals of Internal Medicine*. This study used effect size statistic and demonstrated that OA was associated with a small and clinical irrelevant benefit and an increased risk of adverse events. However, the use of the effect size statistic to infer clinically meaningful changes in efficacy outcomes is frequently misinterpreted. Rutjes *et al*. reported an effect size of 0.37 and then erroneously state that this is equivalent to an improvement in knee pain of 0.9 cm on a 10 cm scale. As showed in Table 1, Rutjes meta-analysis included largest number of RCTs (including published RCTs and grey literature). However, the conclusions in this paper were heavily influenced by inclusion of unpublished, unverifiable data.

The conclusion of the present study is consistent with the finding published in 2015 by Richette and his colleagues^12^. They performed a meta-analysis only including low bias and high-quality RCTs (adequate randomization and concealment and double-blind design) and showed that HA provided a moderate but real benefit for patients with knee OA. Recently, Strand *et al*.^6^ conducted a meta-analysis to investigate the safety and efficacy of UA-approved HA for knee OA. Strand’s study was the only known report to cite the pretreatment to posttreatment standardized mean difference. The statistic results represented very large treatment effects for HA. Thus, it reported that US-approved HA is safe and efficacious through 26weeks in treating knee OA.

The primary limitations of this meta-analysis include the following: (1) English language studies were included in the present overlapping meta-analyses. Although numerous meta-analyses were included in the present study, it is possible that we have omitted non-English language reviews. (2) Several factors of primary trials, such as study design, publication bias and clinical heterogeneity, may influence interpretation. (3) The selected meta-analysis was published in 2006, which will influence the stability of the results. Newest published high-quality meta-analyses are needed to confirm the present evidence.

To sum up, the present systematic review of overlapping meta-analyses investigated efficacy and safety of HA in treating Knee OA. Currently, the best evidence suggested that HA is an effective intervention in treating knee OA without increased risk of adverse events. Therefore, the evidence supports the use of the HA in the treating knee OA. Further studies with effect size statistic are still required to qualify the clinical efficacy.

## Additional Information

**How to cite this article**: Xing, D. *et al*. Intra-articular Hyaluronic Acid in Treating Knee Osteoarthritis: a PRISMA-Compliant Systematic Review of Overlapping Meta-analysis. *Sci. Rep.* **6**, 32790; doi: 10.1038/srep32790 (2016).

## Figures and Tables

**Table 1:** General Description of the Characteristics of included Meta-Analyses.

Authors	Journal	Date of Last Literature search	Date of publication	No. of included studies	No. of included RCTs	No. of grey literature
Lo *et al*.^20^	JAMA	February, 2003	December, 2003	22	19	3
Wang *et al*.^21^	The Journal of bone and joint surgery. American volume	December, 2001	March, 2004	20	20	0
Modawal *et al*.^22^	The Journal of family practice	August, 2004	September, 2005	9	9	0
Arrich *et al*.^23^	CMAJ: Canadian Medical Association journal	April, 2004	April, 2005	22	22	0
Strand *et al*.^24^	Osteoarthritis and cartilage	December, 2004	September, 2006	5	3	2
Bellamy *et al*.^25^	The Cochrane database of systematic reviews	December, 2005	February, 2006	40	40	0
Bannuru *et al*.^9^	Osteoarthritis and cartilage	March, 2010	Jun, 2011	49	49	0
Rutjes *et al*.^10^	Annals of internal medicine	January, 2012	August, 2012	89	71	32
Colen *et al*.^26^	BioDrugs	June, 2011	August, 2012	74	74	0
Miller *et al*.^11^	Clinical medicine insights. Arthritis and musculoskeletal disorders	June, 2013	December. 2013	29	29	0
Richette *et al*.^12^	RMD open	December, 2013	January, 2015	8	8	0
Strand *et al*.^6^	Journal of pain research	December, 2013	May, 2015	29	29	0

**Table 2:** Primary Studies Included in Previous Meta-analyses.

Primary Study	Lo *et al*.^20^	Wang *et al*.^21^	Modawal *et al*.^22^	Arrich *et al*.^23^	Strand *et al*.^24^	Bellamy *et al*.^25^	Bannuru *et al*.^9^	Rutjes *et al*.^10^	Colen *et al*.^26^	Miller *et al*.^11^	Richette *et al*.^12^	Strand *et al*.^6^
Adams *et al*. 1995	−	+	−	−	−	+	−	−	+	−	−	−
Altman *et al*. 1998	+	−	+	+	−	+	+	+	+	+	−	+
Altman *et al*. 2004	−	−	−	−	−	+	+	+	+	−	+	−
Altman *et al*. 2009	−	−	−	−	−	−	+	+	+	+	+	+
Anika *et al*. 2000	−	−	−	−	−	−	−	+	−	−	−	−
Anika *et al*. 2001	−	−	−	−	−	−	−	+	−	−	−	−
Ardic *et al*. 2001	−	−	−	−	−	+	−	+	−	−	−	−
Atamaz *et al*. 2006	−	−	−	−	−	−	−	−	+	−	−	−
Atay *et al*. 2008	−	−	−	−	−	−	−	+	+	−	−	−
Auerbach *et al*. 2002	−	−	−	−	−	+	−	−	+	−	−	−
Baltzer *et al*. 2009	−	−	−	−	−	−	+	+	+	−	−	−
Baraf *et al*. 2009	−	−	−	−	−	−	+	+	−	−	−	−
Bayramoglu *et al*. 2003	−	−	−	−	−	+	−	+	+	−	−	−
Bellamy *et al*. 2005	−	−	−	−	−	−	−	−	+	−	−	−
Bragantini *et al*. 1987	−	+	−	+	−	+	+	+	+	+	−	+
Brandt *et al*. 2001	+	+	−	+	−	+	+	−	+	+	−	+
Bunyaratavej *et al*. 2001	−	−	−	+	−	+	+	+	−	+	−	+
Butun *et al*. 2002	−	−	−	−	−	−	−	+	−	−	−	−
Caborn *et al*. 2004	−	−	−	−	−	+	−	−	+	−	−	−
Caracuel *et al*. 2001	−	−	−	−	−	−	−	+	−	−	−	−
Carrabba *et al*. 1995	+	+	−	+	−	+	+	+	+	+	−	+
Chevalier *et al*. 2010	−	−	−	−	−	−	+	+	+	−	+	−
Chou *et al*. 2009	−	−	−	−	−	−	−	−	+	−	−	−
Cogalgil *et al*. 2002	−	−	−	−	−	−	−	+	−	−	−	−
Cohen *et al*. 1994	+	+	−	−	−	+	+	+	−	−	−	−
Conrozier *et al*. 2009	−	−	−	−	−	−	−	−	+	−	−	−
Corrado *et al*. 1995	+	+	−	+	−	+	+	+	+	−	−	−
Creamer *et al*. 1994	+	+	−	−	−	+	+	+	+	−	−	−
Cubukcu *et al*. 2004	−	−	−	−	−	+	−	−	−	+	−	−
Cubukcu *et al*. 2005	−	−	−	−	−	−	+	+	−	−	−	+
Dahlberg *et al*. 1994	+	−	−	+	−	−	+	−	+	−	−	−
Day *et al*. 2004	−	−	−	+	+	+	+	+	+	+	−	+
DeCaria *et al*. 2012	−	−	−	−	−	−	−	−	−	+	−	+
Dickson *et al*. 1998	−	+	−	−	−	−	−	−	−	−	−	−
Dickson *et al*. 2001	−	−	−	−	−	+	+	+	−	−	−	−
Diracoglu *et al*. 2009	−	−	−	−	−	−	−	+	+	+	−	+
Dixon *et al*. 1988	+	+	−	+	−	+	+	+	+	−	−	−
Dougados *et al*. 1993	+	+	−	+	−	+	+	+	+	−	−	−
Esteve de Miguel *et al*. 1995	−	−	−	−	−	−	−	+	−	−	−	−
Formiguera *et al*. 1995	−	+	−	−	−	+	−	+	−	−	−	−
Forster *et al*. 2003	−	−	−	−	−	+	−	−	+	−	−	−
Frizziero *et al*. 2002	−	−	−	−	−	+	−	−	+	−	−	−
Genzyme *et al*. 2005	−	−	−	−	−	−	−	+	−	−	−	−
Ghirardini *et al*. 1990	−	−	−	−	−	−	−	+	−	−	−	−
Graf *et al*. 1993	−	−	−	−	−	+	−	−	+	−	−	−
Graf von der Schulenburg *et al*. 1997	−	−	−	−	−	−	−	+	−	−	−	−
Grecomoro *et al*. 1987	−	+	+	+	−	+	+	+	+	+	−	+
Groppa *et al*. 2001	−	−	−	−	−	+	−	+	−	−	−	−
Groppa *et al*. 2004	−	−	−	−	−	−	−	+	−	−	−	−
Guler *et al*. 1996	−	−	−	−	−	+	+	+	−	−	−	−
Henderson *et al*. 1994	+	+	+	+	−	+	+	+	+	+	−	+
Heybeli *et al*. 2008	−	−	−	−	−	−	−	+	+	−	−	−
Hizmetli *et al*. 1999	−	−	−	−	−	+	+	−	−	−	−	−
Hizmetli *et al*. 2002	−	−	−	−	−	−	−	+	−	−	−	−
Huang *et al*. 2005	−	−	−	−	−	+	−	+	−	−	−	−
Huang *et al*. 2011	−	−	−	−	−	−	−	+	−	+	−	+
Huskisson *et al*. 1999	+	+	+	+	−	+	+	+	+	+	−	+
Isdale *et al*. 1993	−	−	−	−	−	−	−	+	−	−	−	−
Jorgensen *et al*. 2010	−	−	−	−	−	−	−	+	+	+	−	+
Jubb *et al*. 2003	+	−	−	+	−	+	+	+	+	+	−	+
Juni *et al*. 2007	−	−	−	−	−	−	−	−	+	−	−	−
Kahan *et al*. 2003	−	−	−	−	−	+	−	+	+	−	−	−
Kalay *et al*. 1997	−	−	−	−	−	+	−	+	−	−	−	−
Karatosun *et al*. 2005	−	−	−	−	−	+	−	−	+	−	−	−
Karlsson *et al*. 2002	+	−	−	+	−	+	+	+	+	+	−	+
Kawasaki *et al*. 2009	−	−	−	−	−	−	−	−	+	−	−	−
Kirchner *et al*. 2006	−	−	−	−	−	+	−	−	+	−	−	−
Kosuwon *et al*. 2010	−	−	−	−	−	−	−	+	−	−	−	−
Kotevoglu *et al*. 2006	−	−	−	−	−	−	+	+	+	+	−	+
Kul-Panza *et al*. 2010	−	−	−	−	−	−	−	+	+	+	−	+
Leardini *et al*. 1987	−	−	−	−	−	+	−	−	+	−	−	−
Leardini *et al*. 1991	−	−	−	−	−	+	−	−	+	−	−	−
Lee *et al*. 2006	−	−	−	−	−	−	−	−	+	−	−	−
Lee *et al*. 2011	−	−	−	−	−	−	−	−	+	−	−	−
Leopold *et al*. 2003	−	−	−	−	−	+	−	−	+	−	−	−
Lin *et al*. 2004	−	−	−	−	−	+	−	−	−	−	−	−
Listrat *et al*. 1997	−	−	−	−	−	+	−	+	−	−	−	−
Lohmander *et al*. 1996	+	+	+	+	+	+	+	+	+	+	−	+
Lundsgaard *et al*. 2008	−	−	−	−	−	−	+	+	+	+	+	+
McDonald *et al*. 2000	−	−	−	−	−	+	−	−	−	−	−	−
Miltner *et al*. 2002	−	−	−	−	−	+	−	+	−	−	−	−
Moreland *et al*. 1993	−	−	−	−	−	+	+	+	−	−	−	−
Nahler *et al*. 1996	−	−	−	−	−	+	−	−	−	−	−	−
Nahler *et al*. 1998	−	−	−	−	−	+	−	−	−	−	−	−
Navarro-Sarabia *et al*. 2011	−	−	−	−	−	−	−	+	−	−	+	−
Neustadt *et al*. 2004	−	−	−	−	−	+	−	+	−	−	−	−
Neustadt *et al*. 2005	−	−	−	−	−	+	+	−	+	−	−	−
Onel *et al*. 2008	−	−	−	−	−	−	−	−	+	−	−	−
Ozturk *et al*. 2006	−	−	−	−	−	−	−	−	+	−	−	−
Patrella *et al*. 2002	−	−	−	−	−	−	−	−	+	−	−	−
Pavelka *et al*. 2010	−	−	−	−	−	−	−	+	−	−	−	−
Payne *et al*. 2000	−	−	−	−	−	−	−	+	−	−	−	−
Pedersen *et al*. 1993	−	−	−	−	−	−	−	+	−	−	−	−
Petrella *et al*. 2002	+	−	+	+	−	+	+	+	−	−	−	−
Petrella *et al*. 2006	−	−	−	−	−	−	+	+	+	−	+	
Petrella *et al*. 2008	−	−	−	−	−	−	+	+	+	+	−	+
Petrella *et al*. 2009	−	−	−	−	−	−	−	+	−	−	−	−
Pham *et al*. 2003	+	−	−	−	−	+	−	−	−	−	−	−
Pham *et al*. 2004	−	−	−	−	−	+	+	+	+	−	−	−
Pietrogrande *et al*. 1991	−	−	−	−	−	+	−	−	+	−	−	−
Puhl *et al*. 1993	+	+	+	+	+	+	+	+	+	+	+	+
Raman *et al*. 2008	−	−	−	−	−	−	−	−	+	−	−	−
Raynauld *et al*. 2002	−	−	−	−	−	+	−	+	+	−	−	−
Raynauld *et al*. 2005	−	−	−	−	−	−	−	−	+	−	−	−
Renklitepe *et al*. 2000	−	−	−	−	−	−	−	+	−	−	−	−
Rolf *et al*. 2005	−	−	−	−	−	−	+	−	−	+	−	+
Russell *et al*. 1992	+	−	−	+	−	−	+	+	−	−	−	−
Rydell *et al*. 1972	−	−	−	−	−	−	−	+	−	−	−	−
Sala *et al*. 1995	+	−	−	+	−	−	+	−	−	+	−	+
Sanofi-Aventis *et al*. 2010	−	−	−	−	−	−	−	+	−	−	−	−
Saravanan *et al*. 2002	−	−	−	−	−	−	−	+	−	−	−	−
Scale *et al*. 1994	+	+	+	+	−	+	+	+	+	+	−	+
Schneider *et al*. 1997	−	−	−	−	−	+	−	+	−	−	−	−
Seikagaku *et al*. 2001	−	−	−	−	−	−	−	+	−	−	−	−
Seikagaku *et al*. 2001a	−	−	−	−	−	−	−	+	−	−	−	−
Sezgin *et al*. 2005	−	−	−	−	−	+	+	+	−	−	−	−
Shichikawa *et al*. 1983	−	−	−	−	−	+	+	+	−	−	+	−
Shichikawa *et al*. 1983a	−	−	−	−	−	−	+	+	−	−	−	−
Shimizu *et al*. 2010	−	−	−	−	−	−	−	−	+	−	−	−
Skwara *et al*. 2009	−	−	−	−	−	−	−	−	+	−	−	−
Skwara *et al*. 2009a	−	−	−	−	−	−	−	−	+	−	−	−
Stittik *et al*. 2007	−	−	−	−	−	−	−	−	+	−	−	−
Strand *et al*. 2012	−	−	−	−	−	−	−	−	−	+	−	+
Tamir *et al*. 2001	+	+	−	+	−	+	+	+	+	−	−	−
Tascioglu *et al*. 2003	−	−	−	−	−	+	−	−	+	−	−	−
Tekeoglu *et al*. 1998	−	−	−	−	−	+	−	−	−	−	−	−
Tetik *et al*. 2003	−	−	−	−	−	−	−	+	−	−	−	−
Thompson *et al*. 2002	−	−	−	−	−	+	−	−	−	−	−	−
Tsai *et al*. 2003	−	−	−	−	−	+	+	+	−	−	−	−
Ulucay *et al*. 2007	−	−	−	−	−	−	−	−	+	−	−	−
Vanelli *et al*. 2010	−	−	−	−	−	−	−	−	+	−	−	−
Weiss *et al*. 1981	−	−	−	−	−	−	−	+	+	−	−	−
Weiss *et al*. 1981a	−	−	−	−	−	−	−	+	−	−	−	−
Westrich *et al*. 2009	−	−	−	−	−	−	−	−	+	−	−	−
Wobig *et al*. 1998	+	+	+	+	−	+	+	+	+	+	−	−
Wobig *et al*. 1999	−	−	−	−	−	+	−	−	+	−	−	+
Wu *et al*. 1997	−	+	−	+	−	+	+	+	−	−	−	+
Wu *et al*. 2004	−	−	−	−	−	+	−	+	−	+	−	−

**Table 3:** Databases Mentioned by Included Meta-analyses during Literature Searches.

Authors	Search Database						
	Medline	Embase	Cochrane	BIOSIS	EBSCO	Google Scholar	others
Lo *et al*.^20^	+	−	+	−	−	−	−
Wang *et al*.^21^	+	+	+	−	−	−	−
Modawal *et al*.^22^	+	−	+	−	−	−	−
Arrich *et al*.^23^	+	+	+	+	+	−	−
Strand *et al*.^24^	−	−	−	−	−	−	−
Bellamy *et al*.^25^	+	+	+	−	−	−	+
Bannuru *et al*.^9^	+	+	+	+	+	+	+
Rutjes *et al*.^10^	+	+	+	−	−	−	+
Colen *et al*.^26^	+	+	+	−	−	−	−
Miller *et al*.^11^	+	+	−−	−	−	−	−
Richette *et al*.^12^	+	+	+	−	−	−	−
Strand *et al*.^6^	+	+	−	−	−	−	−

**Table 4:** Methodological Characteristics of Included Meta-analyses.

Authors	Primary study design	Level of evidence	Software	Sensitivity analysis	Subgroup analysis	GARDE evidence profiles
Lo *et al*.^20^	RCT	Level II	SAS	YES	-NO	NO
Wang *et al*.^21^	RCT	Level II	STATA	NO	YES	NO
Modawal *et al*.^22^	RCT	Level II	STATA	YES	NO	NO
Arrich *et al*.^23^	RCT	Level II	STATA	YES	NO	NO
Strand *et al*.^24^	RCT	Level II	SAS	NO	NO	NO
Bellamy *et al*.^25^	RCT	Level II	REVMAN	YES	NO	NO
Bannuru *et al*.^9^	RCT	Level II	R software	YES	YES	NO
Rutjes *et al*.^10^	RCT or quasi-RCT	Level II	STATA	YES	YES	NO
Colen *et al*.^26^	RCT	Level II	REVMAN	NO	NO	NO
Miller *et al*.^11^	RCT	Level II	Comprehensive Meta-analysis	YES	NO	NO
Richette *et al*.^12^	RCT	Level II	R software	NO	NO	NO
Strand *et al*.^6^	RCT	Level II	Comprehensive Meta-analysis	YES	YES	NO

**Table 5:** AMSTAR Criteria for Included Meta-analyses.

Items	Lo *et al*.^20^	Wang *et al*.^21^	Modawal *et al*.^22^	Arrich *et al*.^23^	Strand *et al*.^24^	Bellamy *et al*.^25^	Bannuru *et al*.^9^	Rutjes *et al*.^10^	Colen *et al*.^26^	Miller *et al*.^11^	Richette *et al*.^12^	Strand *et al*.^6^
Was a prior design provided?	0	0	0	0	0	1	0	1	0	0	1	0
Was there duplicate selection and data extraction?	1	1	1	0	0	1	1	1	1	0	1	0
Was a comprehensive literature search preformed?	1	1	1	1	0	1	1	1	1	0	1	1
Was the status of publication used as an inclusion criterion?	1	0	1	1	1	1	1	1	1	0	1	1
Was a list of included/excluded studies provided?	0	0	0	0	0	1	0	0	0	0	0	0
Were the profiles of the included studies provided?	1	1	1	1	1	1	1	1	0	1	1	1
Was the methodological quality of the included studies evaluated and documented?	0	1	0	1	0	1	1	1	0	1	0	1
Was the scientific quality of the included studies used appropriately in formulating conclusions?	0	1	0	1	0	1	1	1	0	0	0	0
Were the methods used to combine the findings of studies appropriate?	1	1	1	1	1	1	1	1	1	1	1	1
Was the publication bias evaluated?	1	1	1	1	0	1	0	1	0	0	1	1
Were the conflicts of interest stated?	1	1	1	1	1	1	1	1	1	1	1	1
Total score	7	8	7	8	4	11	8	10	5	4	8	7

**Table 6:** Heterogeneity of each outcome in included meta-analyses.

Outcomes	Lo *et al*.^20^	Wang *et al*.^21^	Modawal *et al*.^22^	Arrich *et al*.^23^	Strand *et al*.^24^	Bellamy *et al*.^25^	Bannuru *et al*.^9^	Rutjes *et al*.^10^	Colen *et al*.^26^	Miller *et al*.^11^	Richette *et al*.^12^	Strand *et al*.^6^
Overall pooled effect size	+											
Lequesne index score (early)					+	+						
Lequesne index score (late)						+						
Knee function (early)				66%		−	79%			54%	−	54%
Knee function (late)				62%						69%		69%
Knee stiffness							74%					
Physical function								+				
Pain with activities (early)		+				+						
Pain with activities (early)						+						
Pain during or immediately after exercise (early)				81%		+						
Pain during or immediately after exercise (late)				−		−						
Patient global assessment (early)						+						
Patient global assessment (late)						+						
Pain at rest (early)				94%		+						
Pain at rest (late)				−								
Knee pain outcomes (early)			+		−	+	75%	+	92%	73%	32%	73%
Knee pain outcomes (late)			+				32%			75%		75%
WOMAC pain						+						
WOMAC phsical function						+						
Overall adverse events		+		−		−		+		−		−
Flare-ups								+				
Injection-site reaction					−							
Injection-site pain					-							
Arthralgia					−							
Arthropathy/arthrosis/arthritis					−							
Back pain					−							
Headache					−							
Knee effusion								+				
Discontinued due to adverse event					−	−		+				−
Overall study withdrawal						−						−
Mortality		−										

+Has heterogeneity but not reported.

−No heterogeneity.
